# Opposing Roles for CD34 in B16 Melanoma Tumor Growth Alter Early Stage Vasculature and Late Stage Immune Cell Infiltration

**DOI:** 10.1371/journal.pone.0018160

**Published:** 2011-04-11

**Authors:** Steven Maltby, Spencer Freeman, Matthew J. Gold, Jennifer H. E. Baker, Andrew I. Minchinton, Michael R. Gold, Calvin D. Roskelley, Kelly M. McNagny

**Affiliations:** 1 The Biomedical Research Centre, University of British Columbia, Vancouver, Canada; 2 Cellular and Physiological Sciences, University of British Columbia, Vancouver, Canada; 3 Microbiology and Immunology, University of British Columbia, Vancouver, Canada; 4 I3 and CELL Research Groups, University of British Columbia, Vancouver, Canada; 5 Department of Medical Biophysics, British Columbia Cancer Research Centre, University of British Columbia, Vancouver, Canada,; University of California, Los Angeles, and Cedars-Sinai Medical Center, United States of America

## Abstract

Tumor growth and metastasis are determined by the complex interplay of factors, including those intrinsic to tumor cells and extrinsic factors associated with the tumor microenvironment. Our previous work demonstrated key roles for CD34 in the maintenance of vascular integrity and eosinophil and mast cell homing. Since both of these functions affect tumor development, we characterized the effect of CD34 ablation on tumor growth using the B16F1 melanoma model. Intriguingly, we found that CD34 plays a biphasic role in tumor progression. In early growth, both subcutaneous-injected tumors and intravenous-injected lung metastases grew more slowly in *Cd34^−/−^* mice. This correlated with abnormal vessel morphology and increased vascular permeability in these mice. Bone marrow transplantation experiments confirmed that this reflects a non-hematopoietic function of CD34. At later stages, subcutaneous tumor growth was accelerated in *Cd34^−/−^* mice and surpassed growth in wildtype mice. Bone marrow chimera experiments demonstrated this difference was due to a hematopoietic function for CD34 and, correspondingly we found reduced intra-tumor mast cell numbers in *Cd34^−/−^* mice. In aggregate, our analysis reveals a novel role for CD34 in both early and late tumor growth and provides novel insights into the role of the tumor microenvironment in tumor progression.

## Introduction

Cancer results from a complex series of pre-neoplastic genetic lesions in cells that go on to form tumors. Once cells gain tumor-forming potential, their expansion and spread is determined by complex interactions between tumor cells and the surrounding microenvironment. Early growth is governed by proliferation and death of tumor cells and cues from the local microenvironment, resulting in angiogenesis and integration into the local vasculature [1]–[3]. Subsequent growth is affected by tissue remodeling, the supply of pro-tumorigenic factors and evasion of anti-tumor immune responses. Extensive study has focused on initial mutations in carcinogenesis and led to seminal insights into the roles of oncogenes in tumor progression. While these studies provide insight into tumor initiation, a growing body of literature recognizes the importance of the surrounding microenvironment on tumor growth. In this study, we focused on the function of the membrane protein CD34 in the tumor-extrinsic microenvironment.

CD34 is a cell surface sialomucin best known for its expression on hematopoietic stem cell/progenitor cells, and also expressed by vascular endothelia [4], eosinophils [5]–[7] and mast cells [8]. Although CD34 is frequently used to identify progenitor cells, surprisingly little is known about its function. One exception is its role as an L-selectin ligand on the high endothelial venules (HEV), where a specific sialyl Lewis-X modification allows L-selectin binding [9]. However, this modification is limited to the HEV and CD34 function on the vast majority of vasculature and other cell types remains cryptic.

On endothelial cells, CD34 and the related molecule podocalyxin play an important role in vessel development and function [10], [11]. During embryonic vascular development, CD34 and podocalyxin colocalize to sites of lumen formation in the embryonic aorta and adult tumor-associated vessels [10]. Strikingly, *Podxl* ^−/−^ embryos exhibited delayed lumen formation, suggesting that podocalyxin is required for proper vessel formation [10]. A role for endothelial CD34 in maintaining vascular integrity was also proposed in two independent inflammatory models (autoimmune arthritis and TNFα inflamed muscle), as *Cd34^−/−^* mice exhibited increased vascular leakage and edema compared to *Cd34^+/+^* controls [11]. These studies suggest an important role for CD34 and related molecules in vasculogenesis and vessel maintenance.

On hematopoietic cells, we demonstrated a role for CD34 in facilitating mast cell and eosinophil migration. Mast cells derived from *Cd34^−/−^* bone marrow exhibited increased homotypic adhesion and impaired trafficking *in vivo*, compared to *Cd34^+/+^* control cells [6], [12]. *Cd34^−/−^* animals also exhibited reduced tissue eosinophil recruitment in asthma and ulcerative colitis models and *Cd34^−/−^* eosinophils demonstrated a cell-intrinsic reduction in chemotaxis *in vitro* [6], [7].

A variety of hematopoietic cells, including eosinophils and mast cells, which both express CD34, infiltrate tumor sites and interact with tumor cells and the surrounding microenvironment [13], [14]. Local mast cells modulate tumor angiogenesis, tissue remodeling and the host immune response against developing tumors [14]. Initial studies demonstrated a key role for mast cells in promoting angiogenesis in squamous carcinoma and further work showed a similar role in pancreatic tumors [15], [16]. In a colorectal polyp model, we demonstrated the importance of CD34 expression on infiltrating mast cells, resulting in increased tumor angiogenesis in polyp formation [17]. Eosinophils also infiltrate tumors, and may mediate tumor rejection [18], [19]. Since we have previously shown a role for CD34 in both mast cell and eosinophil trafficking, we speculated that CD34 ablation would affect immune cell recruitment into the tumor microenvironment, with direct effects on tumor growth.

In cancer studies, CD34 is often used as a marker of tumor vasculature [20], [21] and CD34^+^ staining is used to characterize vascular patterns within tumor tissues [22]. In the lone study examining a functional role for CD34 in tumor cells, *Cd34^−/−^* mice exhibited reduced tumor growth, compared to wildtype animals, following administration of 7,12-dimethylbenz(a)anthracene (DMBA) and 12-O-tetradecanoylphorbol-13-acetate (TPA) [23]. This growth difference resulted from a decreased capacity of *Cd34^−/−^* hair follicle bulge stem cells (which normally express CD34) to activate and switch to a proliferative state following TPA exposure [23]. These findings demonstrated a cell-intrinsic role for CD34 in follicle stem cell proliferation. However, the function of CD34 in the tumor-extrinsic microenvironment has not been thoroughly examined.

Despite the presence of CD34 on both the vasculature and tumor-infiltrating immune cells, our study is the first to address a role for CD34 in the tumor microenvironment and highlight a tumor cell-extrinsic function for CD34 in tumor development. To assess the function of CD34 on hematopoietic and non-hematopoietic cells we implanted B16F1 melanoma cells into wildtype and *Cd34^−/−^* mice, as well as bone marrow-reconstituted chimeras. Our results show that at an early time-point, tumor growth is decreased in *Cd34^−/−^* animals, in both primary tumors (at day 14) and lung metastases (day 12), and that this is associated with impaired vascular integrity. In contrast, at a later time-point (day 19) tumor growth is increased in *Cd34^−/−^* animals, associated with reduced intra-tumor mast cell numbers.

## Results

### Cd34^−/−^ animals exhibit reduced tumor size at early time-points

**Figure 1:**
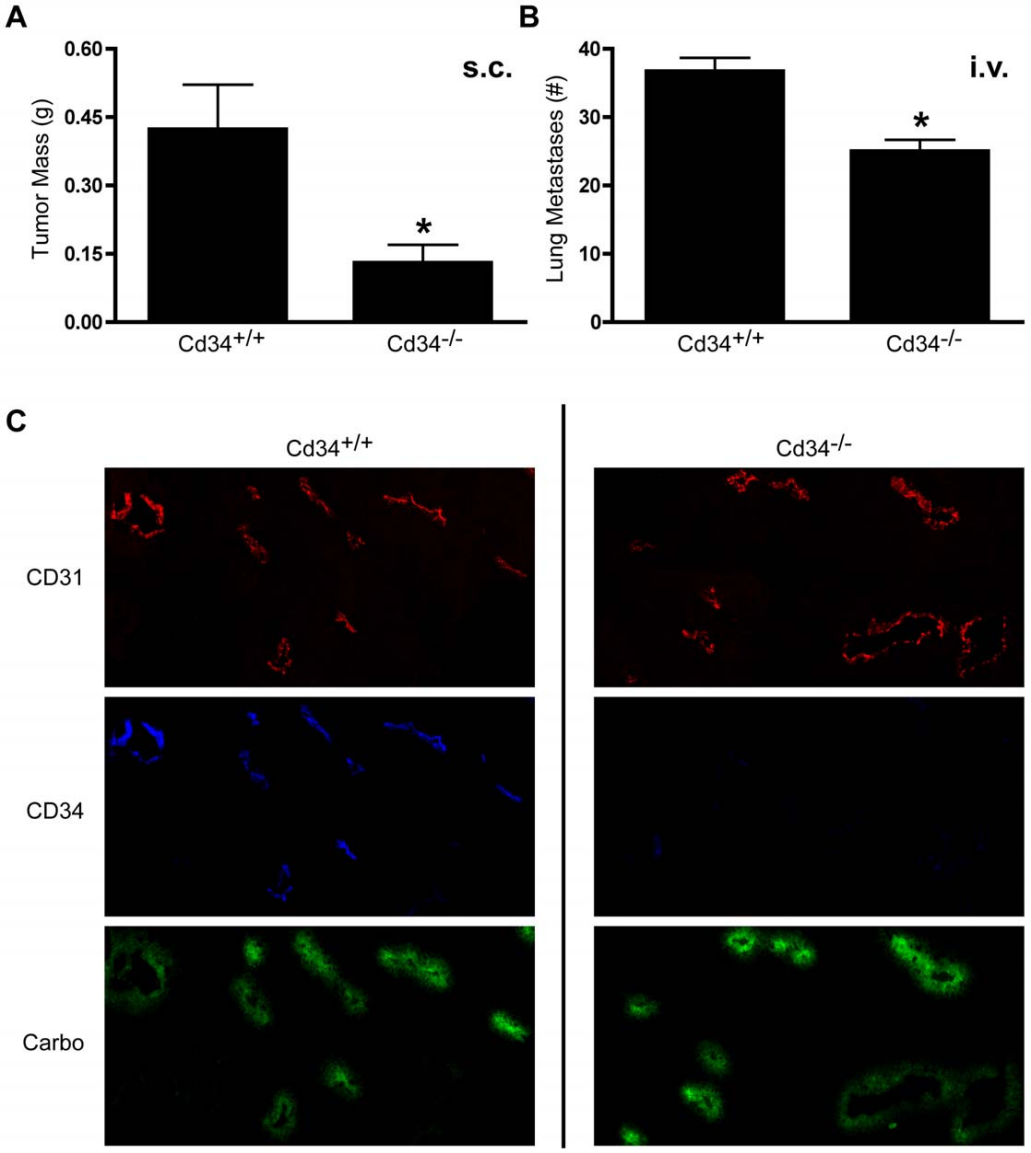
Melanoma growth is reduced in *Cd34^−/−^* animals at day 14, with CD34 expression on vasculature. A, Average tumor mass 14 days after subcutaneous injection of 5×10^5^ B16F1-OVA cells. (Pooled from three experiments, *Cd34^+/+^* n = 12; *Cd34^−/−^* n = 15). B, Average number of lung metastases 12 days after intravenous injection of 3×10^5^ B16F1-OVA cells. (*Cd34^+/+^* n = 5, *Cd34^−/−^* n = 6, *represents p<0.05; Error bars = SEM). C, Representative photomicrographs from s.c.-injected tumor sections stained for CD34 and CD31 or carbocyanine fluorescence, as indicated (CD31-red, CD34-blue, carbocyanine-green).

To determine the effect of CD34 ablation on tumor development and metastasis, B16F1 cells were injected subcutaneously (*s.c.*) or intravenously (*i.v.*) into *Cd34^−/−^* and wildtype *Cd34^+/+^* C57Bl/6 mice. Fourteen days after *s.c.* injection, tumor size was significantly smaller in *Cd34^−/−^* animals, compared to wildtype controls (Figure 1A). Similarly, in the *i.v.* metastasis model, *Cd34^−/−^* mice exhibited fewer metastatic lung nodules 12 days post-injection, compared to wildtype controls (Figure 1B). Thus, CD34 is required for efficient early tumor growth of both *s.c.* and *i.v.*-injected tumors.

As CD34 is expressed on the vascular endothelia, we characterized the expression pattern of CD34 in wildtype tumor tissue, and also confirmed its absence on *Cd34^−/−^* tumor vessels by staining tissue sections with antibodies recognizing CD34. Histological analyses revealed vascular CD34 localization, as previously reported [21], in an overlapping staining pattern with the common vascular marker CD31 (Figure 1C). In tumors excised from *Cd34^−/−^* mice, CD34 staining was absent and CD31 staining patterns were unaffected (Figure 1C). Based on the previous findings of impaired vessel integrity in *Cd34^−/−^* mice [11], we also assayed vascular integrity in tumor tissues by injecting mice with fluorescent carbocyanine dye prior to sacrifice. Interestingly, carbocyanine fluorescence was more intense in *Cd34^−/−^* tumor tissues (Figure 1C), as quantified and discussed in depth below. These findings demonstrate increased vascular dye leakage in *Cd34^−/−^* mice, suggesting a role for CD34 in maintaining tumor vessel integrity and demonstrate that CD34 is required for optimal early tumor growth.

### Reduced tumor size at early time-points is due to loss of CD34 expression on non-hematopoietic cells

**Figure 2:**
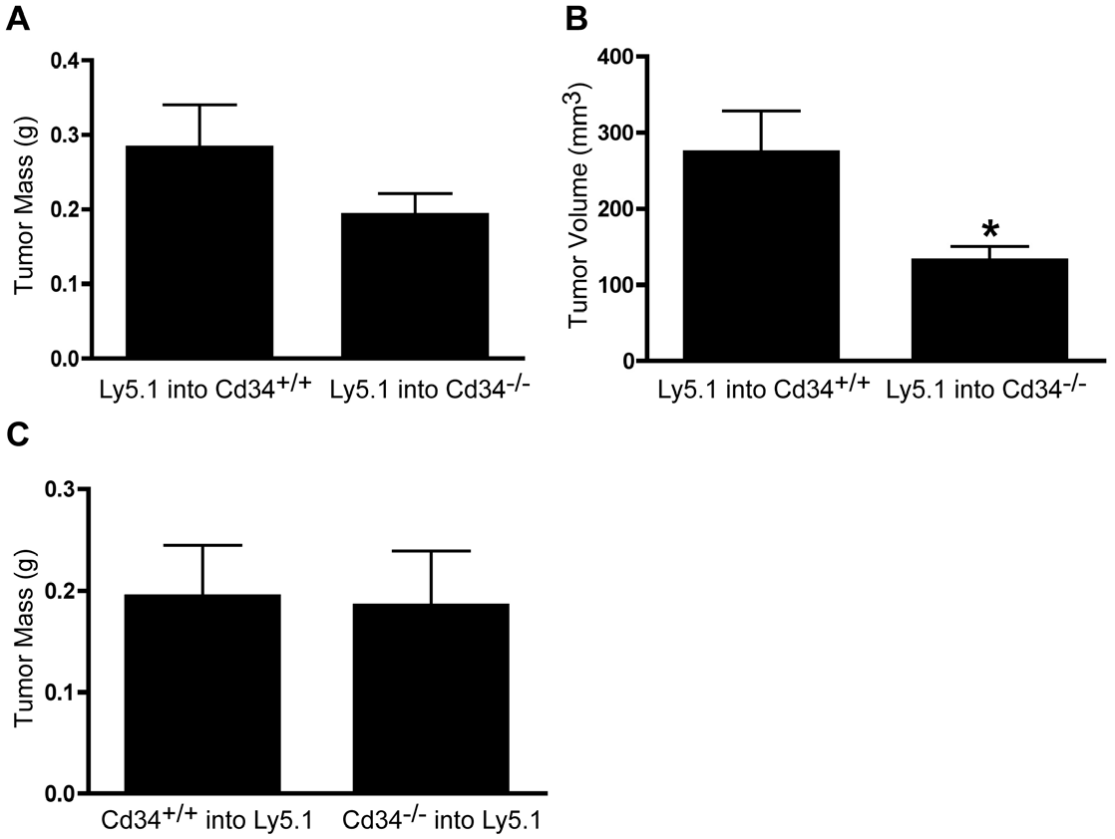
Reduced subcutaneous tumor size at day 14 correlates with loss of CD34 on non-hematopoietic cells. A, Average tumor mass and B, volume at day 14 in *Cd34^+/+^* or *Cd34^−/−^* animals reconstituted with wildtype Ly5.1 bone marrow. (Pooled from two experiments, *Cd34^+/+^* n = 13, *Cd34^−/−^* n = 14). C, Average tumor mass at day 14, in reciprocal reconstitutions of wildtype Ly5.1 animals with *Cd34^+/+^* or *Cd34^−/−^* bone marrow (n = 9, *represents p<0.05; Error bars = SEM).

Several different cell types express CD34, including vascular endothelia and hematopoietic cells (predominantly mast cells and eosinophils). To assess whether loss of CD34 on hematopoietic or non-hematopoietic cells was responsible for the altered tumor growth observed at day 14, we generated bone marrow-reconstituted chimeras. Wildtype and *Cd34^−/−^* animals were irradiated and reconstituted with bone marrow from wildtype Ly5.1 congenic animals. These chimeras retain wildtype CD34 expression on hematopoietic lineages, but lack CD34 on non-hematopoietic tissues. Again, *Cd34^−/−^* animals reconstituted with wildtype bone marrow exhibited reduced tumor size 14 days post-*s.c.* injection, compared to *Cd34^+/+^* animals reconstituted with wildtype bone marrow, as assessed by tumor volume measurements (Figure 2A,B).

To assess the importance of CD34 expression on hematopoietic lineages at the day 14-end-point, we generated reciprocally transplanted chimeras: wildtype Ly5.1 mice were reconstituted with either *Cd34^+/+^* or *Cd34^−/−^* bone marrow. Strikingly, in these chimeras, there was no significant difference in tumor size 14 days post-injection (Figure 2C). These findings demonstrate that CD34 ablation in non-hematopoietic cells results in reduced early *s.c.* tumor growth, while CD34 ablation in hematopoietic cells has no effect at the day 14 time-point.

### Reduced tumor size in Cd34^−/−^ mice is associated with increased vascular permeability and altered vessel structure

**Figure 3:**
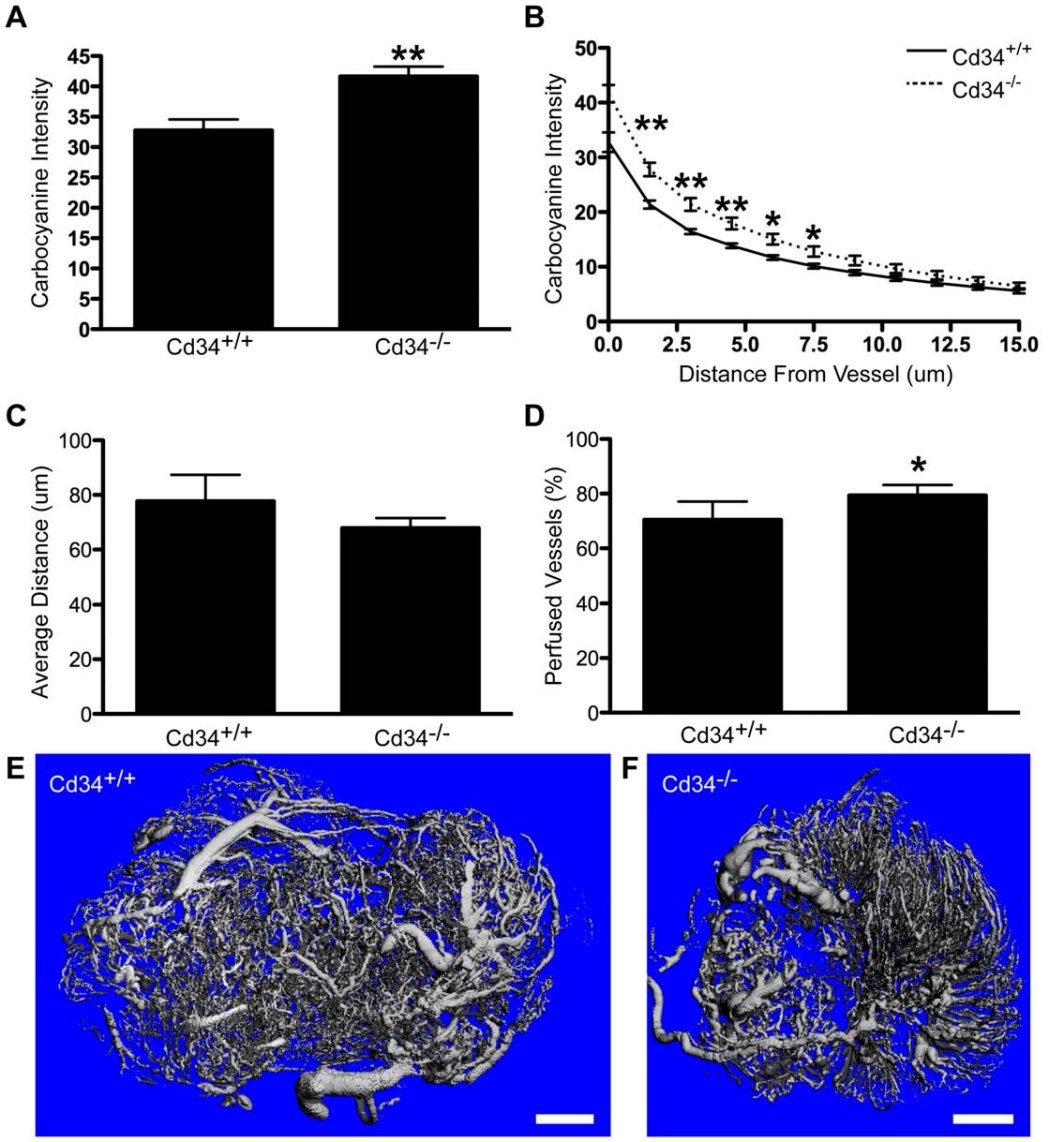
Increased carbocyanine leakage and altered vessel morphology in subcutaneously-injected tumors from *Cd34^−/−^* animals. Carbocyanine fluorescence intensity quantified A, proximal to CD31^+^ vessels (at distance 0 µm) and B, with increasing distance from the nearest vessel (CD31^+^), in day 14 tumors. C, Average distance of each pixel within the tumor tissue from the nearest CD31^+^ staining pixel. D, Perfused vessels (%), corresponding to the percentage of CD31^+^ pixels colocalized with carbocyanine staining. For carbocyanine data, one outlier per genotype was removed prior to analysis. (n = 5 for carbocyanine, n = 6 for average distance, *represents p<0.05; **represents p<0.01; Error bars = SEM). Representative CT images of tumors excised 14 days post-injection and perfused with Microfil contrast reagent from E, *Cd34^+/+^* and F, *Cd34^−/−^* mice. Scale bars, 2 mm (n = 3).

Since the observed difference in *s.c.* tumor growth results from loss of CD34 in non-hematopoietic cells and we have previously shown increased vascular leakage in an inflammatory model [11], we focused our studies on quantifying vascular integrity in tumors. Mice were injected with fluorescent carbocyanine 5 minutes before sacrifice and fluorescence intensity was quantified in tumor sections based on proximity to tumor vasculature. Carbocyanine fluorescence intensity was consistently increased proximal to the vasculature in *Cd34* ^−/−^ tumors, compared to *Cd34^+/+^* tumors, and steadily decreased with increasing distance away from the vessel (Figure 3A,B).

A more detailed quantification of stained sections revealed no difference in vessel density, as assessed by the average distance of each pixel from the nearest CD31^+^ pixel (Figure 3C). Analysis of carbocyanine/CD31 co-localization did reveal a slightly higher proportion of perfused vessels in *Cd34^−/−^* tumor tissues, compared to *Cd34^+/+^*, suggestive of a slight increase in the proportion of functional vessels (Figure 3D). However, further analysis demonstrated no differences in overall tumor vascularity (data not shown), suggesting that while vascular integrity is impaired in *Cd34^−/−^* mice, CD34 ablation has no observable effect on general tumor angiogenesis.

In separate experiments, tumor vasculature was imaged using computed tomography (CT). CT images reveal an organized vascular network in tumors from *Cd34^+/+^* mice, with large vessels of uniform diameter at the tumor periphery and an intricately branched microvascular network throughout the tumor interior (Figure 3E). In contrast, tumors from *Cd34^−/−^* mice consistently exhibited less organized vascular networks, with variability in the shape and size of large vessels, which appeared shorter and more bulbous (Figure 3F). In summary, we observed increased vascular leakage and altered vessel structure in tumors from *Cd34^−/−^* mice, suggesting a role for CD34 in the maintenance of tumor vessel integrity, a limiting factor in tumor growth.

### Cd34^−/−^ mice exhibit increased tumor cell extravasation in a metastasis model

**Figure 4:**
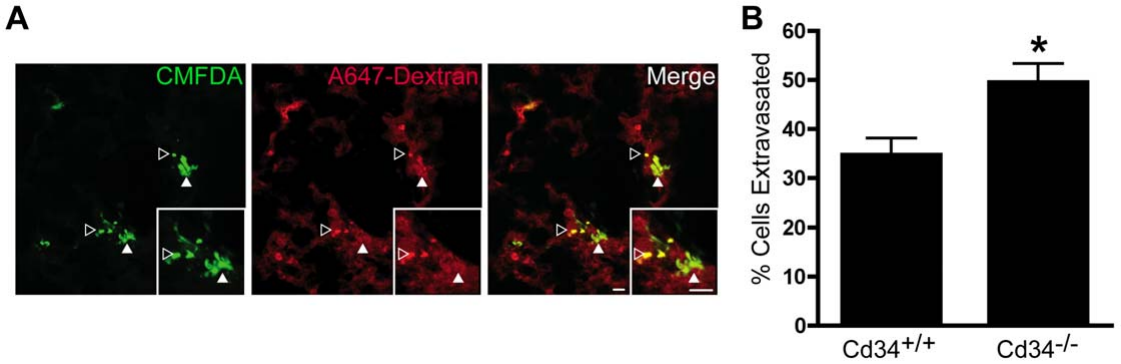
Increased tumor cell extravasation in *Cd34^−/−^* animals. A, CMFDA-labeled B16F1 cells (green) were coinjected *i.v.* with fluorescent dextran (red) and lungs were removed after 4 hours, fixed, sectioned and imaged. Representative sections are shown. Tumor cells were quantified as extravasated (green – solid arrows), or within the microvasculature (yellow – hollow arrows). Scale bar, 50 µm, inset = 2×zoom. B, Proportion (%) of tumor cells extravasated per section (n = 5 lungs per genotype, >5 sections per lung, *represents p<0.05; Error bars = SEM).

Impaired vascular integrity is likely to have several important consequences on tumor cell metastasis including: 1) impaired expansion of existing metastases, due to reduced tumor growth (as observed in primary tumors) and 2) an enhanced ability of tumor cells to initially colonize new sites, due to decreased endothelial junction integrity. To address the latter effect, we revisited our initial metastasis studies to determine the effect of CD34 ablation on initial tumor cell extravasation into the lung. Mice were co-injected with CMFDA-labeled B16F1 cells and fluorescent dextran, to visualize intact vasculature, and lung tissues were extracted after four hours. Fluorescent B16F1 cells were visible within lung sections (Figure 4A - green), with no difference in total tumor cell numbers between *Cd34^+/+^* and *Cd34^−/−^* lungs (data not shown). Fluorescent dextran localization highlighted the lung microvasculature (Figure 4A - red) and increased dextran leakage was seen in *Cd34^−/−^* lungs, compared to *Cd34^+/+^* lungs. Merged images (Figure 4A) were used to quantify the proportion of total tumor cells that migrated into the lung parenchyma, revealing increased tumor cell extravasation in *Cd34^−/−^* lungs, compared to *Cd34^+/+^* controls (Figure 4B). These findings suggest that the impaired vessel integrity observed in *Cd34^−/−^* tumor vasculature also occurs in the lung microvasculature, resulting in an increased initial cell extravasation in *Cd34^−/−^* lungs. However, despite enhanced initial colonization in the lung, the later requirement for CD34 to maintain vascular integrity and promote tumor growth is dominant and CD34 ablation results in fewer detectable metastases at the day 12 time-point (Figure 1B).

### Cd34^−/−^ animals exhibit a paradoxical increased tumor growth at later time-points, as a result of CD34 loss on hematopoietic cells

**Figure 5:**
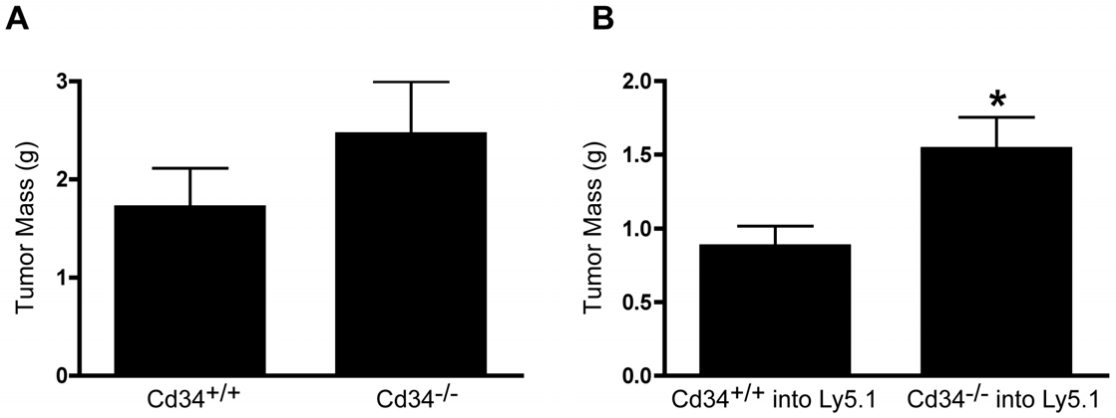
Tumor size in *Cd34^−/−^* surpasses *Cd34^+/+^* by day 19, due to hematopoietic loss of CD34. A, Average *s.c.-*injected tumor masses 19 days post-injection in *Cd34^+/+^* and *Cd34^−/−^* animals. (Pooled from three experiments, *Cd34^+/+^* n = 15, *Cd34^−/−^* n = 16). B, Average tumor mass at day 19 in wildtype Ly5.1 animals reconstituted with either *Cd34^+/+^* or *Cd34^−/−^* bone marrow. (Pooled from two experiments, *Cd34^+/+^* into Ly5.1 n = 11, *Cd34^−/−^* into Ly5.1 n = 13). (*represents p<0.05; Error bars = SEM).

As tumor size was significantly reduced 14 days post-injection, we hypothesized that this difference would increase at later time-points. To address this hypothesis, tumors in *Cd34^−/−^* animals were weighed 19 days post-injection. Between day 14 and day 19, tumors masses increased dramatically from ∼0.4 grams to >1.0 gram in wildtype mice. Surprisingly, tumor size in *Cd34^−/−^* animals approached or even surpassed tumor size in *Cd34^+/+^* control animals, at the day 19 end-point (Figure 5A).

To determine the relative importance of CD34 expression on hematopoietic versus non-hematopoietic cells at this time-point, tumor cells were, again, implanted into bone marrow chimeric animals. Ly5.1 mice reconstituted with *Cd34^−/−^* bone marrow exhibited increased tumor size on day 19, compared to mice reconstituted with *Cd34^+/+^* bone marrow (Figure 5B). These findings suggest that, at late stages, CD34 expression on hematopoietic cells limits tumor growth and that CD34 ablation in hematopoietic cells results in increased tumor growth. As hematopoietic CD34 is predominantly expressed on mast cells and eosinophils, we further examined the roles of these cells in regulating tumor growth.

### Cd34^−/−^ intra-tumoral mast cell infiltration is impaired

**Figure 6:**
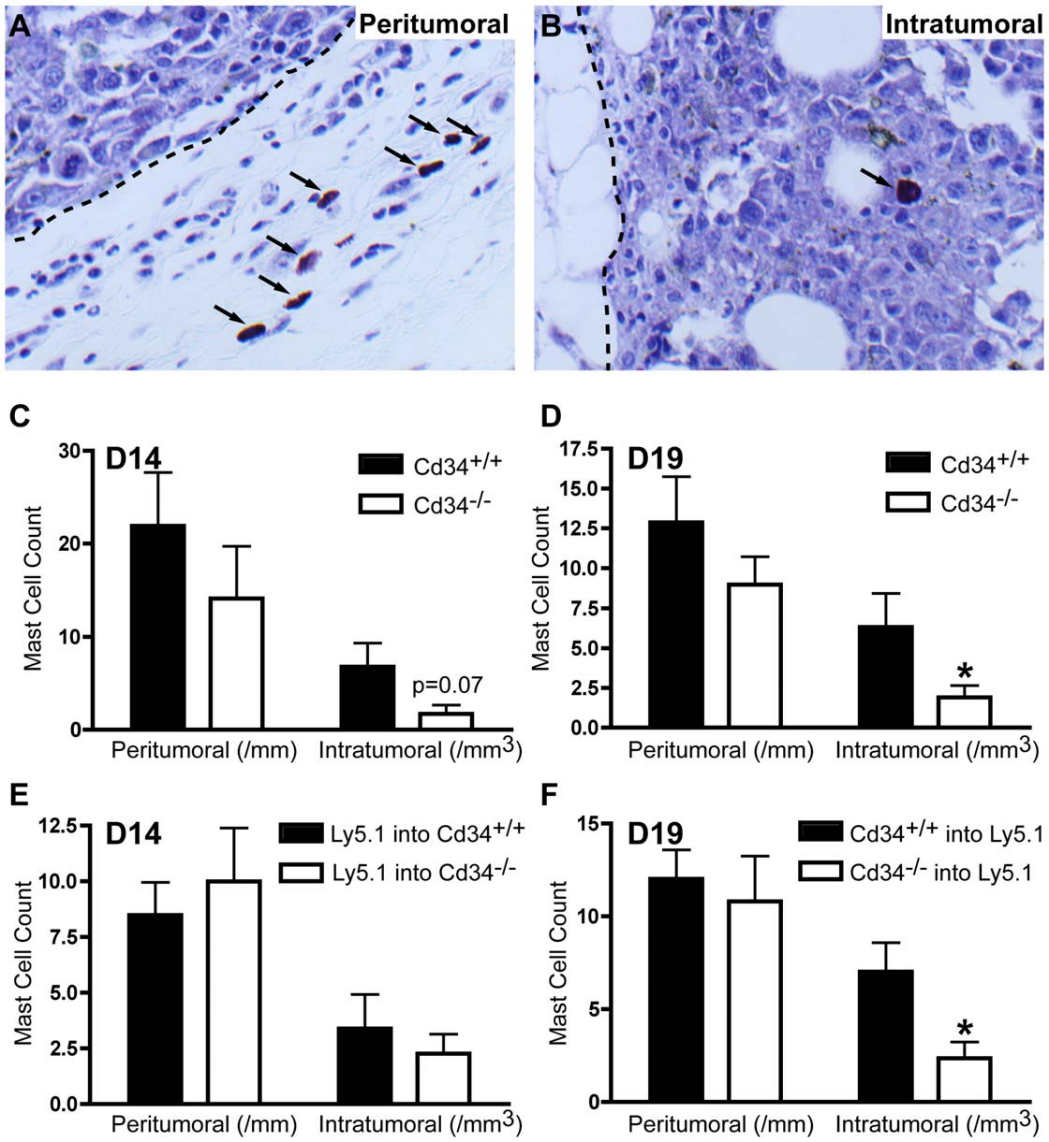
Mast cell tumor infiltration is reduced in *Cd34^−/−^* mice. Toluidine blue staining of A, peri-tumoral mast cells in tissues surrounding tumors and B, intra-tumoral mast cells within tumor tissues. (arrows, mast cells; dotted line, tumor boundary). Mast cell counts in *s.c.-*injected tumors from *Cd34^+/+^* and *Cd34^−/−^* animals at C, day 14 (pooled from three experiments, *Cd34^+/+^* n = 13, *Cd34^−/−^* n = 16) and D, day 19 (pooled from three experiments, n = 13). Counts in tumors from E, *Cd34^+/+^* or *Cd34^−/−^* animals reconstituted with wildtype Ly5.1 marrow at day 14 (pooled from three experiments, Ly5.1 into *Cd34^+/+^* n = 13, Ly5.1 into *Cd34^−/−^* n = 14) and F, in wildtype Ly5.1 animals reconstituted with either *Cd34^+/+^* or *Cd34^−/−^* bone marrow at day 19 (pooled from three experiments, *Cd34^+/+^* into Ly5.1 n = 11, *Cd34^−/−^* into Ly5.1 n = 13). (*represents p<0.05; Error bars = SEM).

Mast cells play many roles in tumor development, with functions in both tumor promotion and suppression [14]. Further, the localization of mast cell populations within and around developing tumors can have opposing effects on tumor growth [24]. Since we previously showed that CD34 is required for efficient mast cell homing and migration [6], [12], we quantified the number of mast cells in tumors from *Cd34^+/+^* and *Cd34^−/−^* mice. Toluidine blue staining revealed mast cell accumulation around the periphery of developing tumors, with the majority of mast cells positioned in healthy tissues directly adjacent to tumors (peri-tumoral; Figure 6A) and a small number of mast cells penetrating into tumor tissue (intra-tumoral; Figure 6B).

At the early (day 14) time-point, peri-tumoral and intra-tumoral mast cell numbers were slightly reduced (p = 0.07) in tumors from *Cd34^−/−^* animals, compared to *Cd34^+/+^* controls (Figure 6C), with the reduction in intra-tumoral mast cell numbers being significant at the day 19 time-point (Figure 6D). In tumors from chimeric animals, chimeras lacking CD34 on non-hematopoietic tissues exhibited no difference in mast cell infiltration at the day 14 time-point (Figure 6E). Conversely, at day 19, chimeras lacking hematopoietic CD34 expression exhibited reduced intra-tumoral mast cell numbers, compared to chimeras reconstituted with wildtype bone marrow (Figure 6F). These findings reveal reduced mast cell tumor infiltration at all time-points when CD34 is ablated on hematopoietic lineages, most significantly at late stages, resulting in decreased numbers of intra-tumoral mast cells.

## Discussion

CD34 expression in the tumor microenvironment has been extensively reported in human and mouse cancers. Notably, CD34 expression is generally absent in melanocytic tumor samples [22] and in the current studies, we found no significant CD34 staining on murine melanoma cells (Figure 1C). Thus any role for CD34 in tumor progression is likely restricted to a tumor cell-extrinsic function in the tumor microenvironment. In this regard, across a broad range of tumor types, CD34 is expressed on tumor vasculature and on spindle cells in a variety of skin lesions [22]. Within these lesions, CD34 expression has been used diagnostically [22] and has been proposed as a prognostic indicator, as CD34 staining correlates with the extent of tumor angiogenesis [25]. In mouse models, CD34 staining has also been used to quantify microvessel density [20], [21]. To our knowledge, however, the functional significance of the CD34 molecule itself, on the tumor vasculature, has not been addressed.

We previously demonstrated a role for endothelia-expressed CD34 in the maintenance of vessel integrity in a murine model of rheumatoid arthritis [11]. *Cd34^−/−^* mice exhibited increased vascular leakage in distal joints in response to autoimmune serum (leading to worsened disease outcomes) and following TNFα treatment [11]. The loss of the CD34-related molecule, podocalyxin, also delays aortic lumen formation during early embryogenesis [10]. Further, podocalyxin and CD34 both localize to the site of vascular lumen initiation in Lewis Lung carcinoma tumor-associated vasculature [10]. Thus, although the function of this family of proteins on tumor vasculature has never been directly addressed, there is a precedent to suggest a role for these molecules in both vessel formation and the maintenance of vascular integrity.

**Figure 7:**
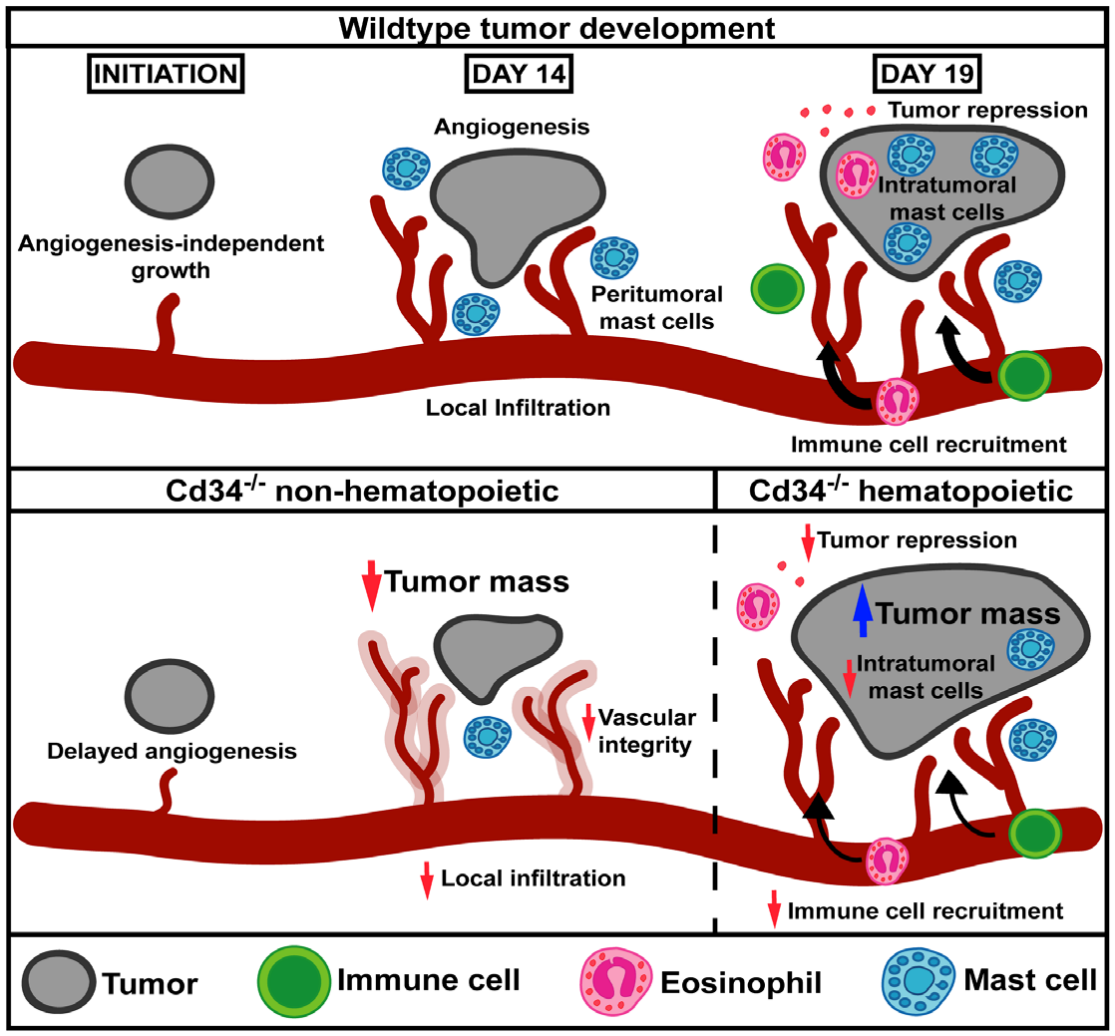
CD34 function in the tumor microenvironment. Model schematic of CD34 function in the tumor microenvironment, highlighting effects on vascular integrity, associated with decreased tumor growth at an early time-point (day 14) and immune cell accumulation, associated with increased tumor growth at a later time-point (day 19) in *Cd34^−/−^* mice.

In the current study, we observed biphasic functional roles for CD34 in the tumor microenvironment during tumor progression (Figure 7). At an early time-point (day 14), loss of CD34 on non-hematopoietic cells affected tumor vasculature resulting in decreased tumor size, increased vascular leakage and altered vessel morphology. Reduced numbers of lung metastases were also observed in *Cd34^−/−^* mice at day 12, despite increased initial tumor cell extravasation into the lung 4 hours after injection. All these phenotypes support a role for CD34 on vascular endothelial cells, consistent with the findings of a role for the CD34 family of proteins in maintaining vascular integrity in both inflammatory disease and embryonic development [10], [11].

Although we detect impaired vessel integrity at an early time-point, we observed no significant differences in vessel density (Figure 3C) or vascularization between *Cd34^+/+^* and *Cd34^−/−^* tumors, suggesting that impairment may occur early in tumor growth or may be quite subtle. In support of the second hypothesis, we found increased tissue carbocyanine leakage, altered vessel morphology and increased tumor cell extravasation in the lungs of *Cd34^−/−^* mice, compared to wildtype animals. These findings support the conclusion that CD34 is involved in the maintenance or establishment of vessel integrity within the tumor microenvironment. In light of these findings, extra care should be taken when evaluating CD34 expression as the sole marker of tumor vasculature. Based on our findings, we predict that if CD34 expression is lost or reduced on tumor vasculature, there would be functional effects on vascular integrity and tumor growth. As such, multiple vascular markers should be used to ensure all tumor vasculature is recognized and further studies should be performed to determine whether CD34^−^ vasculature is present in different tumor subsets.

It should be noted that the reduced tumor mass at day 14 in reconstituted *Cd34^−/−^* animals (Figure 2A/B) was less severe than the reduction in non-irradiated, non-reconstituted *Cd34^−/−^* animals (Figure 1A). This suggests that while CD34 ablation, specifically on non-hematopoietic cells, results in impaired tumor growth, CD34 expression on hematopoietic cells may also play a lesser role. In non-reconstituted *Cd34^−/−^* mice we observed reduced mast cell numbers, which was not observed in tumors from *Cd34^−/−^* mice reconstituted with *Cd34^+/+^* bone marrow (Figure 6C,E). Mast cells are thought to promote tumor growth by inducing angiogenesis and remodeling the tumor microenvironment in certain situations [14]. In fact, mast cell-deficient W/W^v^ mice exhibit decreased early tumor growth in a B16 melanoma model [26]. We propose that endothelial-expressed CD34 is most important for early tumor growth by maintaining vascular integrity, but the reduction in mast cell accumulation in *Cd34^−/−^* mice may also alter angiogenesis and play a minor role at the early time-point (day 14).

At the later time-point (day 19), we found that tumor size is increased in *Cd34^−/−^* mice, following CD34 ablation in hematopoietic cells (Figure 7). CD34 expression on mature hematopoietic cells occurs predominantly on mast cells and eosinophils. Intra-tumoral mast cell infiltration was reduced at all time-points following *Cd34* ablation in hematopoietic cells and, while mast cells have been suggested to play pro-tumorigenic roles, studies have also indicated a role for mast cells in anti-tumoral immunity [24]. In addition to mast cells, eosinophils play a role in tumor rejection in B16 tumors [18], [19], [27]. Our previous studies have demonstrated a key role for CD34 in optimal eosinophil migration *in vivo* and *in vitro* [6], [7] and we speculate that increased tumor growth at the late time-point in *Cd34^−/−^* mice may also be due in part to impaired eosinophil migration, resulting in reduced tumor clearance. Thus we propose that the impaired intra-tumoral mast cell and eosinophil infiltration, in *Cd34^−/−^* mice, results in decreased tumor clearance and increased tumor growth at the late time-point.

In conclusion, our study demonstrates that CD34 promotes tumor growth at an early time-point (day 14), by playing a key role on vasculature, maintaining appropriate vessel integrity. In *Cd34^−/−^* animals at all time-points, intra-tumoral mast cell numbers are reduced, and at a later time-point (day 19) tumor growth surpasses *Cd34^+/+^* controls. The difference at the later time-point can be attributed to CD34 loss on hematopoietic cells and likely reflects a reduced capacity of *Cd34^−/−^* mast cells and eosinophils to dampen tumor growth and induce immune tumor rejection. This study presents novel insights into CD34 function on vasculature and expands our understanding of a role for CD34 in hematopoietic cell migration during tumor development.

## Materials and Methods

### Ethics Statement

All experiments and procedures were approved by the Committee on Animal Care (Protocol #A07-0258 and #A07-0399) at the University of British Columbia, in accordance with the requirements of the Canadian Council on Animal Care (CCAC).

### Mice

*Cd34^−/−^* (provided by Dr. T. W. Mak. [28]) and C57Bl/6 mice were used for all experiments. Six to ten week old gender-matched mice were maintained in specific pathogen-free conditions at The Biomedical Research Centre.

### Bone marrow reconstitutions

Bone marrow chimeras were generated using the Ly5.1/5.2 reconstitution system, as previously described [6]. Bone marrow was isolated from either donor *Cd34^+/+^* (Ly 5.2) and *Cd34^−/−^* (Ly 5.2) or Ly5.1 mice and transplanted into recipient Ly5.1 or *Cd34^+/+^* and *Cd34^−/−^* mice respectively, as specified in the text. Cells (∼10^6^) were intravenously injected into lethally-irradiated (11 Gy) recipient animals. After ten weeks, reconstitution levels were evaluated by assessing Ly 5.2 and Ly 5.1 expression in blood and mice were considered reconstituted when hematopoietic cells were >80% donor-derived.

### Subcutaneous and lung metastasis tumor induction

B16F1-OVA cells (provided by Dr. C. Parrish [18]) were maintained in RPMI supplemented with 10% FBS, penicillin, streptomycin and L-glutamine. For subcutaneous models, 5×10^5^ cells were injected into the fore flank and tumors grown for 14 to 19 days, as indicated in the text. Tumor size was quantified by measuring final tumor mass and tumor volume using manual calipers (calculated as (*L*×*W* ^2^)/2). Excised solid tumors for H&E and toluidine blue histology were fixed overnight in 10% buffered formalin and paraffin-embedded. For immunohistochemistry studies, tumors were excised and frozen at −20 C, then embedded in OCT (Tissue-TEK). Lung metastases were induced by intravenous injection of 3×10^5^ B16F1-OVA cells and mice were sacrificed after 12 days to quantify lung tumor numbers.

### Histology

To assess tumor mast cell numbers, toluidine blue staining was performed on fixed tumor sections. Mast cells were counted in tissue bordering tumors (peri-tumoral) and within tumor tissue (intra-tumoral) and normalized to tumor circumference and tumor area respectively. Slides were analyzed on a Zeiss Axioplan2 microscope (Toronto, ON) and images were captured using a Qimaging Retiga EX CCD camera (Minneapolis, MN) and Openlab 4.0.4 software.

Immunohistochemistry was performed as previously described [29], [30]. Five minutes prior to euthanization, mice were intravenously administered 0.6 mg/mL fluorescent carboycyanine (Molecular Probes), dissolved in 75% (v/v) DMSO: 25% sterile water. Tissue sections were prepared 2–3 mm from the tumor edge, dried and imaged for fluorescence, then fixed in 50% (v/v) acetone/methanol. Tissues were stained using antibodies recognizing CD31 (MEC 13.3, BD PharMingen) and CD34 antibody (RAM34, BD PharMingen). Tissues were imaged using a robotic fluorescence microscope (Zeiss Imager Z1), a cooled, monochrome CCD camera (Retiga 4000R, Qimaging), a motorized slide loader and x-y stage (Ludl Electronic Products) and customized NIH-ImageJ software. Images were inverted, overlaid and cropped, with staining artifacts removed. Carbocyanine intensity was assessed by sorting pixels based on relative distance from CD31-positive vessels, reported in 1.5 µm increments, after subtracting background-staining intensity. Vessel density was assessed by measuring the relative distance of each pixel from the nearest CD31^+^ pixel and vessel perfusion was assessed by colocalization of carbocyanine fluorescence with CD31 staining.

### Computed tomography (CT) imaging

Mice were anaesthetized with ketamine/xylazine, injected subcutaneously with heparin (500 U; Sigma, St. Louis, MO) and perfused with warmed PBS followed by a solidifying silicone rubber contrast agent (Microfil MV-122; Flow Tech, Carver, MA). Mice were kept on ice for two hours for curing, after which tumors were excised and stored in 10% EDTA for two weeks for decalcification. Tumors were scanned on a micro-CT imaging system (vivaCT 40, Scanco Medical, Bassersdorf, Switzerland) at 45 kVp, 177 µA with a 10.5 µm voxel size. All images were prepared at the same threshold values

### Tumor extravasation studies

2.5×10^5^ B16F1 cells (American Type Culture Collections), labeled with CellTracker Green dye (CMFDA; Molecular Probes, Invitrogen), were coinjected intravenously with 5 mg/kg AlexaFluor647-dextran (Molecular Probes, Invitrogen), as previously described [31]. After four hours, lungs were removed, fixed in paraformaldehyde, embedded in OCT, sectioned and imaged using the UPlan Fluorite 20×/0.7 NA objective of an Olympus FV1000 confocal microscope. Five or more sections were analyzed for each lung sample.

### Statistical analysis

*P* values were calculated using unpaired two-sample student *t* tests.
